# The effect of histo-blood group ABO system transferase (BGAT) on pregnancy related outcomes：A Mendelian randomization study

**DOI:** 10.1016/j.csbj.2024.04.040

**Published:** 2024-04-29

**Authors:** Yuqi Sun, Haonan Zheng, Manqing Wang, Rongrong Gu, Xueyan Wu, Qian Yang, Huiling Zhao, Yufang Bi, Jie Zheng

**Affiliations:** a Department of Endocrine and Metabolic Diseases, Shanghai Institute of Endocrine and Metabolic Diseases, Ruijin Hospital, Shanghai Jiao Tong University School of Medicine, Shanghai, China; b College of Health Science and Technology,Shanghai Jiao Tong University School of Medicine, China; c Shanghai National Clinical Research Center for metabolic Diseases, Key Laboratory for Endocrine and Metabolic Diseases of the National Health Commission of the PR China, Shanghai Key Laboratory for Endocrine Tumor, Shanghai Digital Medicine Innovation Center, Ruijin Hospital, Shanghai Jiao Tong University School of Medicine, Shanghai, China; d College of Basic Medical Science,Shanghai Jiao Tong University School of Medicine, China; e MRC Integrative Epidemiology Unit (IEU), Bristol Medical School, University of Bristol, Oakfield House, Oakfield Grove, Bristol BS8 2BN, United Kingdom

**Keywords:** Mendelian randomization, ABO, BGAT, venous complication in pregnancy, gestational hypertension

## Abstract

Protein level of Histo-Blood Group ABO System Transferase (BGAT) has been reported to be associated with cardiometabolic diseases. But its effect on pregnancy related outcomes still remains unclear. Here we conducted a two-sample Mendelian randomization (MR) study to ascertain the putative causal roles of protein levels of BGAT in pregnancy related outcomes. *Cis*-acting protein quantitative trait loci (pQTLs) robustly associated with protein level of BGAT (P < 5 ×10^−8^) were used as instruments to proxy the BGAT protein level (N = 35,559, data from deCODE), with two additional pQTL datasets from Fenland (N = 10,708) and INTERVAL (N = 3301) used as validation exposures. Ten pregnancy related diseases and complications were selected as outcomes. We observed that a higher protein level of BGAT showed a putative causal effect on venous complications and haemorrhoids in pregnancy (VH) (odds ratio [OR]=1.19, 95% confidence interval [95% CI]=1.12–1.27, colocalization probability=91%), which was validated by using pQTLs from Fenland and INTERVAL. The Mendelian randomization results further showed effects of the BGAT protein on gestational hypertension (GH) (OR=0.97, 95% CI=0.96–0.99), despite little colocalization evidence to support it. Sensitivity analyses, including proteome-wide Mendelian randomization of the *cis*-acting BGAT pQTLs, showed little evidence of horizontal pleiotropy. Correctively, our study prioritised BGAT as a putative causal protein for venous complications and haemorrhoids in pregnancy. Future epidemiology and clinical studies are needed to investigate whether BGAT can be considered as a drug target to prevent adverse pregnancy outcomes.

## Graphical Abstract

**Figure:**
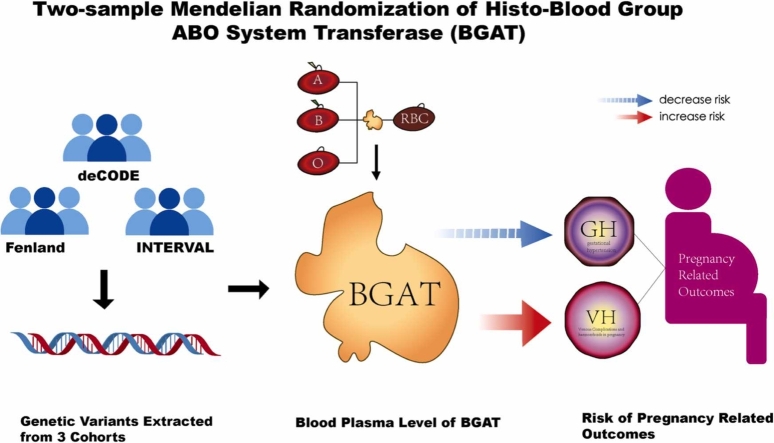

## 1 Introduction

Adverse pregnancy outcomes are health conditions related to both pregnant women and newborns that always arose concerns in our society. Recent studies indicated that preterm labor is a leading cause of perinatal morbidity and mortality in developed countries [1]. Pregnancy induced venous complications includes pregnancy related deep vein thrombosis and lower limb varicose veins, causes lower limb swelling, pain, skin ulcers, and difficulties for the daily activities (e.g. sleep), and even threatens lives of pregnant women [2]. Hemorrhoids can be considered as a common venous disease that has a significant impact on life quality of pregnant women [3]. Gestational hypertension is a major cause of maternal, fetal and newborn morbidity and mortality. Women with pregnancy-induced hypertension syndrome are at higher risks of abruptio placentae, cerebrovascular events, organ failure and disseminated intravascular coagulation [4]. Despite the frequent occurrence of these uncomfortable symptoms during pregnancy, effective prevention methods or ideal treatment options are still limited [5], [6]. Identification of genetic regulators that influence pregnancy related outcomes will help better understand the etiology of these conditions, and may support the development of drug targets to reduce risks of these outcomes.

The ABO blood group system categorizes human blood into four main types: A, B, AB, and O, based on the presence or absence of specific antigens on the surface of red blood cells. Several studies observed the associations of ABO blood group types with pregnancy related outcomes, such as stillbirth [7] and obstetrical hemorrhage risk [8]. Yet many of these results are not consistent [9]. Moreover, the evidence supporting the association between ABO blood group system and pregnancy related outcomes is still limited, requiring large birth cohorts or record-linkage data. Instead, BGAT (i.e. Histo-Blood Group ABO System Transferase) is a blood group related biomarker. One recent study had showed that the activity of BGAT in plasma was related to a higher risk of venous thromboembolism (VET) risk [10], which may inform intervention targets for cardiovascular diseases. Inspired by these findings, we considered plasma BGAT levels as an attractive blood group related biomarker, informing potential intervention targets for pregnancy related vascular complications.

Given it would not be feasible to conduct a randomized control trail among pregnant women due to ethical concerns [11], Mendelian randomization (MR) provides an alternative way to assess the impact of BGAT protein level on pregnancy outcomes. Two-sample MR is a genetic epidemiology method using germline genetic variants (also called single nucleotide polymorphisms (SNPs)) as instruments to estimate the causal effect of an exposure on an outcome [12]. Previous MR studies identified effects of protein level of BGAT on many diseases, including COVID-19 [13] and ischemic stroke [14]. However, its causal relationships with pregnancy related outcomes remain unclear. Recently available large-scale genome-wide association studies (GWASs) of protein level of BGAT [15], [16], [17] and adverse pregnancy outcomes [18], [19], [20] provided a timely opportunity to investigate the causal effect of the BGAT protein level on those outcomes using MR.

The aim of this study was to estimate the causal efffects of protein level of BGAT with ten pregnancy related outcomes using two-sample MR. To increase reliability of the findings, the causal estimates were validated in three independent BGAT datasets (deCODE [15], Fenland [16] and INTERVAL [17]). To further increase the possibility of identifying true causality, we applied a sensitivity analysis pipeline [21], including Steiger filtering [22], and genetic colocalization [23], [24], [25]. Given pleiotropic potential of the BGAT instruments, we applied a proteome-wide MR to further investigate the possibility of horizontal pleiotropy.

## 2 Materials and methods

### 2.1 Genetic instruments selection of BGAT protein levels

**Fig. 1:**
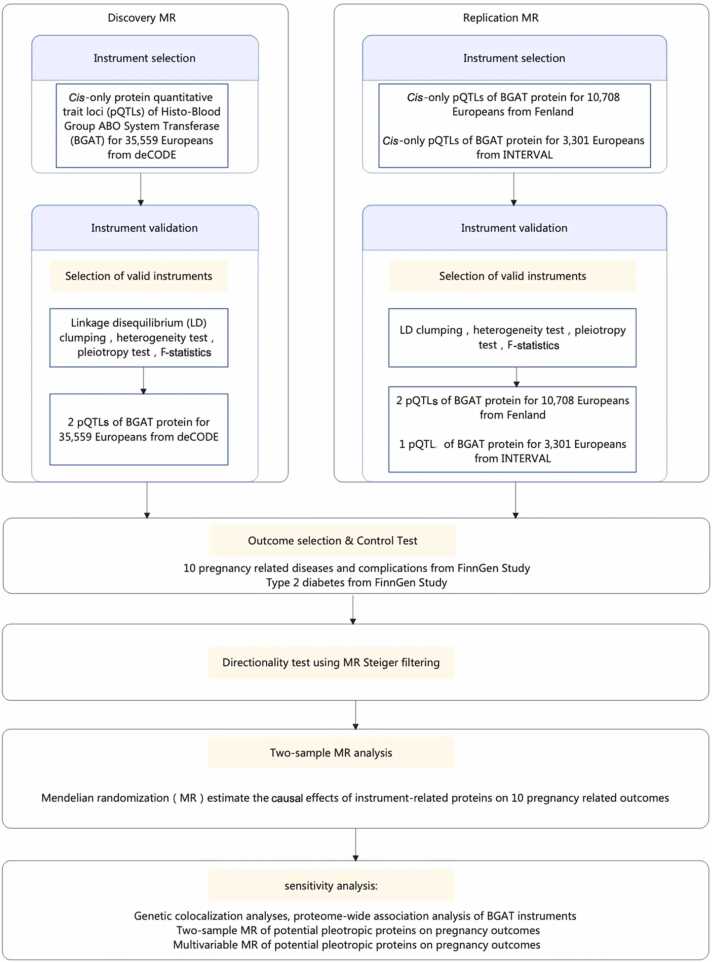
**Study design of two-sample Mendelian randomization exploring effects of BGAT protein with pregnancy outcomes** Abbreviations: pQTL, protein quantitative trait loci; LD, linkage disequilibrium; T2D, type 2 diabetes.

Fig. 1 presented the study design. *Cis*-acting protein quantitative trait loci (pQTLs) from three proteome GWAS studies were used as candidate instruments for the discovery (deCODE n = 35,559) and validation (Fenland n = 10,708 and INTERVAL n = 3301) MR analyses. All three proteome GWAS studies utilized SOMAscan assay to measure plasma protein levels of BGAT, a technology takes advantage of the dual nature of nucleic acid ligands: target recognition through shape complementarity and highly specific hybridization to complementary sequences arrayed on surfaces [26]. The deCODE and Fenland study used SOMAscan 4.0 with a total of 5000 protein measurements while INTERVAL study used SOMAscan 3.0 with 3000 protein measurements. Given multiple conditional *cis*-acting pQTLs exist in the ABO region after linkage disequilibrium (LD) clumping, all these *cis*-acting pQTLs were treated as genetic instruments for BGAT. The following criteria were applied for the instrument selection: 1) only *cis*-acting pQTLs (defined as genetic variants ≤500 kb from the leading pQTL within the BGAT region) were considered in this study since *cis*-acting pQTLs are more likely to have direct biological functions. *Trans*-acting pQTLs of BGAT were excluded due to their possible pleiotropic effects; 2) we selected pQTLs that were robustly associated with the protein level of BGAT (P-value threshold < 5 ×10^−8^) and with relatively strong instrument strength (F-statistics>10) ^27^; 3) LD clumping was conducted to satisfy the independent assumption of instruments (threshold of LD r^2^ <0.2 for exclusion of highly correlated pQTLs). Given potential weak LD between pQTL instruments, we applied generalized inverse variance weighted (gIVW) which increased number of *cis*-acting pQTLs included in the model, while still taking into account the weak LD correlations among pQTLs. 4) The strength of genetic instruments was evaluated using F-statistic, with an F-statistic> 10 suggesting a strong instrument [27]. After selection, three groups of *cis*-acting pQTLs (two from deCODE [**Table S1**]; two from Fenland [**Table S2**]; one from INTERVAL [**Table S3**]) were used as instruments for the subsequent MR analyses (LD matrix among instruments were listed in **Table S4**). Instruments from deCODE were selected for discovery analyses since it has the largest sample size among the three datasets, and the other two were used for validation analyses.

### 2.2 Genetic associations of pregnancy outcomes

A total of ten pregnancy related diseases and complications were selected as outcomes for this study, including venous complications and hemorrhoids in pregnancy, gestational hypertension, pre-eclampsia, infections of genitourinary tract in pregnancy, gestational oedema and proteinuria without hypertension, preterm labor and delivery, spontaneous abortion, gestational diabetes mellitus and other maternal disorders predominantly related to pregnancy (**Table S5**). Type 2 diabetes (T2D) was selected as a positive control outcome since its association with protein level of BGAT has already been confirmed (**Table S6**) [28]. To avoid sample overlap with pQTL GWAS, we obtained GWAS summary statistics (including estimates of regression coefficient, the corresponding standard error and P value, effect allele, other allele, and effect allele frequency) for all the outcomes from FinnGen [20].

### 2.3 Steiger filtering

With large sample sizes of the exposure and outcome GWAS data, we are likely to identify genetic variants robustly associated with both the exposure and outcomes. Therefore, we may observe reverse causality of genetic variants that influence the outcome first and then influence the exposure as a consequence. To minimise the possibility of reverse causality, we performed Steiger filtering [22] to select valid genetic instruments that showed a right direction of exposure-outcome effect. Steiger filtering assumes that a valid instrument should explain more variance of the exposure than that of the outcome, and identifies those instruments that explained more variance of the outcome than that of the exposure for removal from MR analyses.

### 2.4 MR analysis

We applied two-sample MR to estimate the effect of protein level of BGAT on pregnancy outcomes (β_XY_). Wald ratio estimate [29], i.e., the ratio of the genetic association of the outcome (β_GY_) against the genetic association of the exposure (β_GX_), was used to estimate the effect based on each instrument. The gIVW with multiplicative random effects [30], was applied to combine the Wald ratio estimates for multiple variants for the same exposure. We conducted the discovery MR by using genetic instruments from deCODE, and re-conducted gIVW by using those from Fenland and INTERVAL for validation. Since these instruments are obtained from three independent European-based cohorts, the reliability of our MR findings will increase if consistent results are shown across those datasets. To control for multiple testing, we used Bonferroni correction to calculate the false discovery rate (Bonferroni-corrected P-value≤0.05) for robust results.

### 2.5 Colocalization analysis

Genetic colocalization analysis was conducted to detect whether the identified MR signals shared causal variants between the protein expression of BGAT and pregnancy outcomes, or were just due to confounding by LD. It is a method used to detect whether two traits (such as a disease and a biomarker) share the same genetic variants. In this study, we applied colocalization analysis for those effect with MR evidence. This is because (1) MR was employed here to discover potential causal effects of BGAT levels on pregnancy outcomes, while the colocalization analysis was designed to deal with the similar causal question as a sensitivity analysis, validating and enhancing the reliability of the MR findings; (2) BGAT-outcome association with MR evidence can be confounded by LD (which means an identified MR effect was observed due to another variant in LD with the instruments independently influenced the outcome), where colocalization can distinct causality from confounding by LD. Therefore, a positive colocalization finding typically implies a non-zero Mendelian randomization estimate, the reverse is not generally true [31]. Under the assumption of a single causal variant within each genomic region, the Bayesian statistical framework quantifies the posterior probability of association (PPA) for each of the four possible hypotheses [32] in validation analysis (H1/H2: the genetic variants [i.e. pQTLs] were only associated with the protein level of BGAT or associated with a pregnancy outcome in the tested genomic region; H3: there were genetic variants associated with both protein expression of BGAT and a pregnancy outcome in the tested genomic region but the two traits did not shared the same causal variant; H4: there were genetic variants associated with both protein expression of BGAT and a pregnancy outcome, and the two traits shared the same causal variant in the tested genomic region). The effect estimates and allele information for all variants within (500KB window upstream or downstream of the leading pQTL for each trait were extracted for the colocalization analysis. We assigned prior probabilities that a variant is associated with trait 1 (p_1_ = 1 ×10^−4^), trait 2 (p_2_ = 1 ×10^−4^), and both traits (p_12_ = 1 ×10^−5^) as recommended. The PP H4/(PP H3 + PP H4) ≥ 90% was considered as the threshold for colocalization tests. We also conducted an LD check analysis between BGAT pQTLs and the outcome that had showed evidence for colocalization using the above Bayesian method, to further validate the results. The LD check window was set up as ± 500 kb, and LD r^2^ ≥ 0.8 was considered as evidence for colocalization [21].

### 2.6 Proteome-wide association analysis of BGAT instruments and MR of potential pleiotropic proteins on pregnancy outcomes

It is known that the BGAT pQTLs were pleiotropic [17], which means a BGAT *cis* signal could be a *trans* signal for other proteins. To evaluate the influence of their horizontal pleiotropy, we performed a two-step proteome-wide MR approach using the instruments from deCODE. First, we searched for the genetic effects of each BGAT instrument across all publicly available proteome-wide GWAS on IEU OpenGWAS [18], [19] to identify its effect on other proteins. These were potential pleiotropic proteins that may link BGAT instruments with outcomes not via BGAT protein expression. Then we selected those proteins that were associated with our instruments as exposures, and used MR method to check whether there were causal relationships between those potential pleiotropic proteins and our pregnancy outcomes. All pQTLs associated with these potential pleiotropic proteins were used as instruments for this analysis. To control for multiple testing, we used the conservative Bonferroni-adjusted P-value (i.e.P ≤ 0.05) to define robust MR estimates. Where a pleiotropic protein identified for an outcome, we further conducted a multivariable MR to obtain the direct effect of BGAT protein on the outcome adjusting for the pleiotropic protein [33].

In this study, MR analyses and Steiger filtering tests were performed using the TwoSampleMR package, and colocalization analysis were conducted using the coloc package. Results were visualized using the ggplot2 and LocusZoom [34] in the R software platform (version 4.2.2; R Development Core Team).

## 3 Results

We reported findings according to the STROBE-MR (Strengthening the Reporting of Mendelian Randomization Studies) guidelines [35], [36](**Supplementary Form1**).

### 3.1 Summary of BGAT instrument selection

**Fig. 2:**
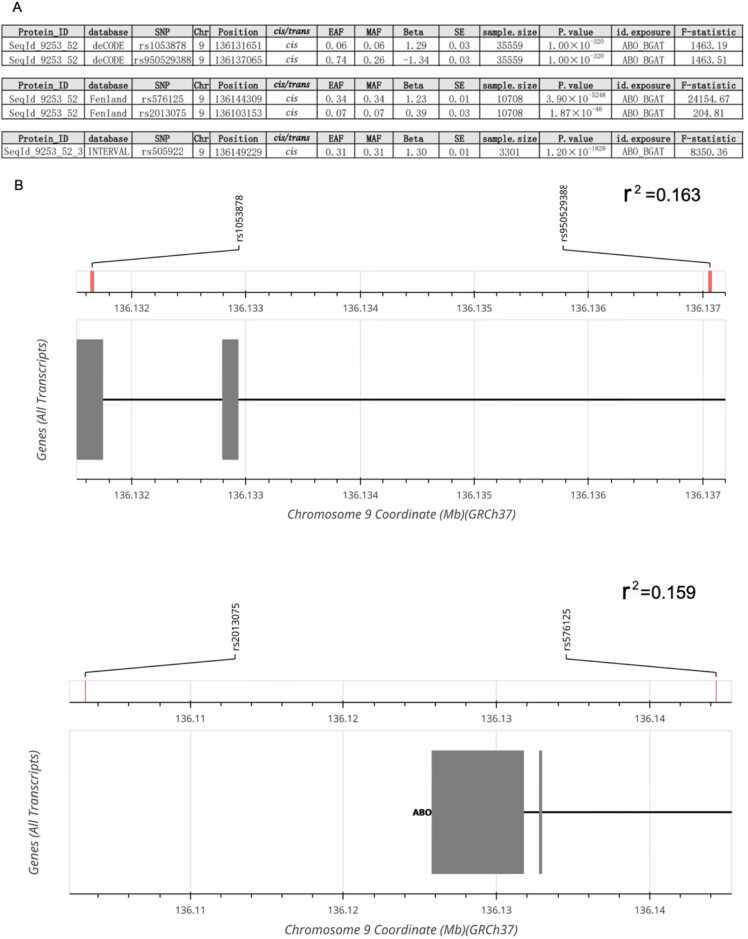
**Information of the*****cis*****-pQTLs selected as instruments to represent BGAT protein levels** (A) Genetic association information of the instruments been selected in the three proteome GWAS studies. (B) The pair-wise LD correlation and location of the selected instruments extracted from deCODE and Fenland. Abbreviations: Chr, chromosome; EAF, effect allele frequency; MAF, minor effect allele frequency; SE, standard error; r [2], the LD correlation of the instruments.

We selected two instruments (rs1053878 and rs950529388) associated with protein level of BGAT that reached the genome-wide significance (P-value < 5 × 10^−8^) in the discovery cohort (i.e. deCODE). The instrument strength of a combination of these two instruments were strong (total F-statistic =2926.69), where each of the individual instrument also showed a strong strength (**Table S1**). The two BGAT instruments were weakly correlated with each other (LD r [2] < 0.2), and were used in the discovery MR analyses (**Table S4A**). Other two sets of instruments (two instruments selected from Fenland and one instrument selected from INTERVAL) were used to proxy BGAT protein level in the validation MR analyses (**Table S2** and **S3**). All the five selected instruments were common variants with a minor allele frequency > 0.05, passed the Steiger filtering test of directionality (**Table S7**) and were not palindromic SNPs with minor allele frequencies larger than 0.42. Fig. 2**A** presented the results of instruments selection. Fig. 2**B** presented the location of each instrument and the LD clumping results from LDmatrix application in the NIH Ldlink website, version 5.6.5 [37]. Table 2 presented the detailed protein assay technology information and cohort difference of deCODE, Fenland and INTERVAL.

### 3.2 Effect of protein levels of BGAT on pregnancy related outcomes using deCODE data

**Fig. 3:**
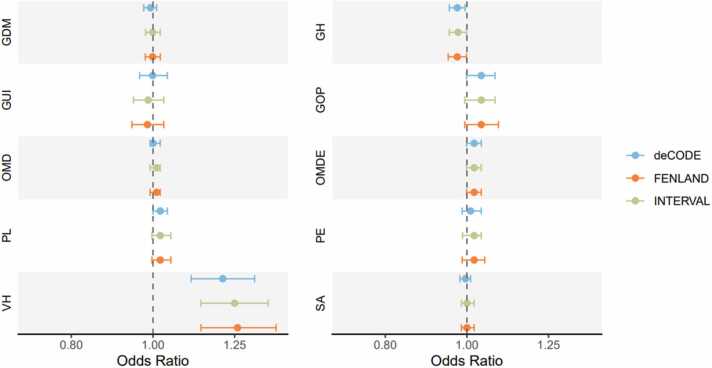
**Two-sample Mendelian randomization estimates for associations of BGAT protein with ten pregnancy outcomes** Abbreviations: GDM, gestational diabetes mellitus; GH, gestational [pregnancy-induced] hypertension); GUI, infections of genitourinary tract in pregnancy; GOP, gestational [pregnancy-induced] oedema and proteinuria without hypertension; OMD, other maternal disorders predominantly related to pregnancy; OMDE, other maternal diseases classifiable elsewhere but complicating pregnancy, childbirth and the puerperium; PL, preterm labour and delivery; PE, pre-eclampsia; VH, venous complications and haemorrhoids in pregnancy; SA, spontaneous abortion.

**Fig. 4:**
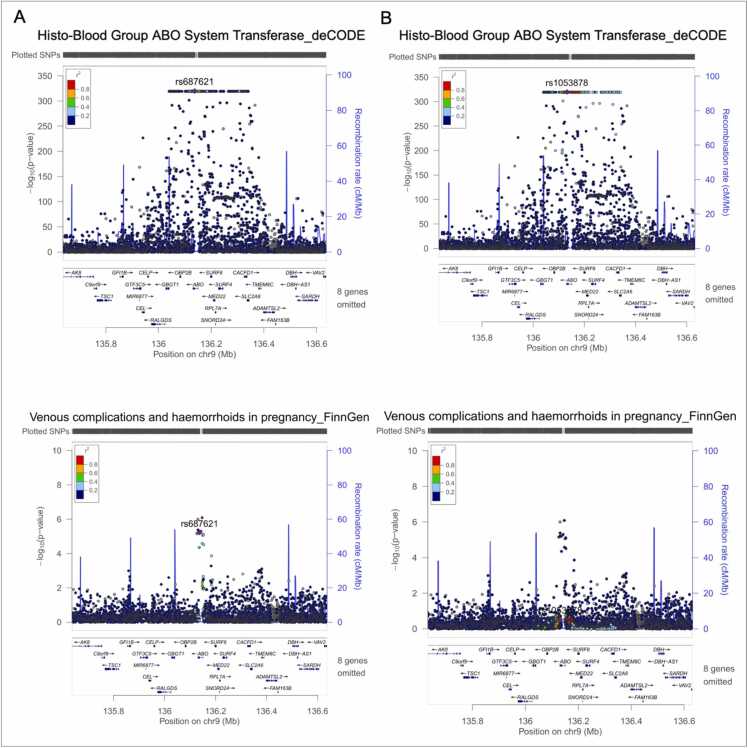
**Regional plots of BGAT pQTLs – venous complications and haemorrhoids in pregnancy with Mendelian randomization evidence of potential causality** (A) Regional plots of rs950529388 (merged into rs687621). (B) Regional plots of rs1053878.

Table 1 and Fig. 3 present two-sample MR estimates for associations of BGAT pQTLs in deCODE with pregnancy-related outcomes. In the discovery analysis, MR suggested that higher protein levels of BGAT showed a putative causal effect on increased risks of pregnancy induced venous complications and haemorrhoids in pregnancy (OR=1.19; 95% CI: 1.12–1.27; Bonferroni-corrected P-value =8.26 ×10^−7^) (Fig. 3). Strong evidence of colocalization indicated that venous complications and haemorrhoids in pregnancy (colocalization probability=91%) shared the same causal variants with the protein level of BGAT (Table 1). The MR also suggested that higher protein levels of BGAT showed a putative causal effect on decreased GH (OR=0.97; 95% CI: 0.96–0.99; Bonferroni-corrected P-value =0.03) (Fig. 3), yet with little supporting evidence of colocalization (colocalization probability=12%) (Table 1). The MR analyses for other pregnancy related outcomes did not reach the Bonferroni threshold, and their colocalization analyses further suggested little evidence of causal effects on those outcomes. Through the LD check between BGAT pQTLs and venous complications, we identified that one SNP (rs687621, merged with our instrument - rs950529388) in deCODE was strongly correlated with the leading SNP in the outcome dataset (LD r^2^ >0.8), suggesting a potential causal relationship. LD check for the other instrument (rs1053878) did not show such a relationship (Fig. 4).

**Table 1:** Two-sample MR estimates for associations of BGAT protein with ten pregnancy outcomes using instruments from deCODE, Fenland and INTERVAL.

	**Discovery MR**		**Replication MR**					**Colocalization using Bayesian method**^c^				
**Outcome**	**deCODE**^a^		**Fenland**^a^			**INTERVAL**^b^		**H1**	**H2**	**H3**	**H4**	**H4/** **(H3+H4)**
	**OR** **(95% CI)**	**Bonferroni** **P-val**	**OR** **(95% CI)**	**Bonferroni** **P-val**		**OR** **(95% CI)**	**Bonferroni** **P-val**					
VH	1.19 (1.12-1.27)	8.26×10^-7^	1.27 (1.15-1.40)	1.71×10^-5^		1.25 (1.14-1.37)	4.78×10^-5^	2.68×10^-^^3^	0	8.71×10^-^^2^	0.91	0.91
GH	0.97 (0.96-0.99)	0.03	0.97 (0.95-1.00)	0.39		0.98 (0.95-1.00)	0.45	0.68	0	0.29	0.04	0.12
OMDE	1.02 (1.00-1.03)	0.18	1.02 (0.99-1.04)	0.76		1.02 (1.00-1.04)	0.64	0.76	0	0.21	0.03	0.13
GOP	1.04 (1.01-1.07)	0.19	1.04 (0.99-1.08)	1.00		1.04 (0.99-1.08)	0.97	0.69	0	0.27	0.04	0.13
PL	1.02 (1.01-1.04)	0.07	1.02 (1.00-1.05)	0.76		1.02 (1.00-1.05)	0.80	0.75	0	0.22	0.03	0.12
Pre-eclampsia	1.01 (0.99-1.03)	1.00	1.02 (0.99-1.05)	1.00		1.02 (0.99-1.04)	1.00	0.61	0	0.38	9.48×10^-^^3^	0.02
GUI	1.01 (0.98-1.03)	1.00	0.99 (0.95-1.03)	1.00		0.99 (0.95-1.03)	1.00	0.62	0	0.38	9.76×10^-^^3^	0.03
OMD	1.00 (0.99-1.01)	1.00	1.00 (0.99-1.02)	1.00		1.01 (0.99-1.02)	1.00	0.80	0	0.20	4.85×10^-^^3^	0.02
Spontaneous abortion	0.99 (0.98-1.01)	1.00	1.00 (0.98-1.02)	1.00		1.00 (0.99-1.02)	1.00	0.76	0	0.23	4.57×10^-^^3^	0.02
GDM	0.99 (0.98-1.00)	1.00	1.00 (0.98-1.02)	0.10		1.00 (0.98-1.02)	1.00	0.73	0	0.26	4.83×10^-^^3^	0.02
Positive control												
Type 2 diabetes	1.04 (1.03-1.04)	3.58×10^-18^	1.05 (1.03-1.06)	3.43×10^-16^		1.04 (1.03-1.06)	2.09×10^-16^	6.54×10^-1^^3^	0	4.65×10^-^^2^	0.95	0.95

Abbreviations: GDM, gestational diabeties mellitus; GH, gestational [pregnancy-induced] hypertension; GOP, gestational [pregnancy-induced] oedema and proteinuria without hypertension; GUI, infections of genitourinary tract in pregnancy, MR, Mendelian randomization, OMD, other maternal disorders predominantly related to pregnancy, OMDE, other maternal diseases classifiable elsewhere but complicating pregnancy, childbirth and the puerperium, OR, odds ratio; PL, preterm labour and delivery,VH, venous complications and haemorrhoids in pregnancy.

a Two SNPs were used as instruments in MR generalized inverse variance weighted method to obtain the estimates.

b One SNP was used as the instrument in MR Wald ratio method to obtain the estimates.

c We used 5867 SNPs in the colocalization analyses.

### 3.3 Effect of protein levels of BGAT on pregnancy related outcomes using Fenland and INTERVAL data

In Table 1 and Fig. 3, BGAT pQTLs were consistently associated with venous complications and haemorrhoids in pregnancy using data from Fenland and INTERVAL (OR=1.27; 95% CI: 1.15–1.40; Bonferroni-corrected P-value=1.71 ×10^−5^, OR=1.25; 95% CI: 1.14–1.37; Bonferroni-corrected P-value=4.78 ×10^−5^, respectively). These estimates were also consistent with findings from the discovery analysis, suggesting the robustness of the causal relationship between BGAT protein and venous complications. Consistent with the discovery MR, associations of BGAT pQTLs with other pregnancy related outcomes did not reach the P-value threshold after Bonferroni correction here (Table 1 and Fig. 3). .

**Table 2:** The protein assay and technology of deCODE, Fenland and INTERVAL:.

Cohort	Ancestry Group	Protein Assay Technology	Version	Protein measurements
deCODE	European	SOMAscan	4.0	5000
Fenland	European	SOMAscan	4.0	5000
INTERVAL	European	SOMAscan	3.0	3000

### 3.4 Positive control analysis of BGAT protein levels on T2D

A higher level of BGAT was associated with an increased risk of T2D using data from deCODE (OR=1.04, 95% CI: 1.03–1.04, P-value=3.58 ×10^−18^), Fenland (OR=1.05, 95% CI: 1.03–1.06, P-value=3.43 ×10^−16^) and INTERVAL (OR=1.04, 95%CI: 1.03–1.06, P-value=2.09 ×10^−16^). Colocalization analysis also supports the causal effect of BGAT pQTLs on T2D (colocalization probability=95%; Table 1).

### 3.5 Proteome-wide association study of BGAT instruments and MR of potential pleiotropy proteins on pregnancy related outcomes

To estimate the influence of horizontal pleiotropy on the MR findings, we first looked up the genetic associations of BGAT instruments on all available proteome GWASs listed in the IEU OpenGWAS database (prot-a, prot-b and prot-c; **Table S8** and **S9**). We found that the two BGAT instruments from deCODE (i.e.rs1053878 and rs950529388 (merged into rs687621)) were associated with 108 proteins in total, besides BGAT. For these 108 proteins, we extracted their instruments and conducted two-sample MR of these proteins on our pregnancy related outcomes. We found that none of these proteins was associated with venous complications and haemorrhoids in pregnancy with Bonferroni-adjusted P-values < 0.01, which implies that unbalanced horizontal pleiotropy is less likely to be an issue for our MR results. We also identified that two proteins (toll-like receptor 4: lymphocyte antigen 96 complex and lactase-phlorizin hydrolase) were associated with GDM and GH, respectively (Bonferroni-adjusted P-values <0.01, **Table S10**). Using multivariable MR to adjust for these two proteins, we found little evidence for the association of BGAT protein with GDM or GH (**Table S11** and **S12**), which were consistent with our univariate MR results in Table 1.

## 4 Discussion

In this study, we estimated the causal relationships of BGAT protein levels on ten pregnancy related outcomes using two-sample MR, and validated the findings in three independent datasets of BGAT pQTLs (deCODE as discovery, Fenland and INTERVAL as validation). Our study, for the first time, showed that one standard deviation higher of BGAT protein level was associated with an increased risk of pregnancy induced venous complications and haemorrhoids in pregnancy, while the other 55 proteins showed little evidence for an effect with this outcome. Sensitivity analyses including genetic colocalization and proteome-wide MR of potential pleiotropic instruments further strength the possibility of identifying true causal effects in this finding.

### 4.1 The effect of pregnancy related VH on fetus and neonates

Our findings may have clinical impacts on fetus and neonates too. The plenty hormonal, hemodynamic and mechanical changes occurred in pregnancy make women vulnerable to suffer from venous complications and hemorrhoids. For fetus and neonates, maternal chronic venous disease is associated with an increased inflammatory milieu and significant damage in maternofetal tissues, such as the umbilical cord [38]. Previous study further suggested that for women with portal vein hypertension during pregnancy, miscarriages occurred in 14% (95% CI: 8–20%) of them, preterm birth in 27% (95% CI: 19–37%), and low birth weights in 22% (95% CI: 15–30%) [39]. Our finding identified the effect of BGAT on venous complications and hemorrhoids which might provide an intervention targets to reduce these pregnancy complications and further reduce risks of serious health issues for fetuses and neonates.

### 4.2 Comparison with previous studies

In order to compare our top findings with previous literature, we comprehensively reviewed both conventional observational studies and MR studies estimating associations of BGAT protein with any health outcomes. However, we did not identify any specific articles exploring an effect of circulating BGAT protein on pregnancy related diseases or symptoms. Most existing studies focused on ABO blood group types, and found some associations with not only pregnancy outcomes (discussed below), but also outcomes in general population (e.g. cardiovascular diseases [40], knee osteoarthritis [41], stroke [42], thrombosis [43], [44], [45] and T2D [46]).

Our novel finding for the positive association of BGAT protein expression in plasma with venous complications and haemorrhoids in pregnancy might not be directly compared to the previous studies investigating ABO blood group among the general population. Previous studies consistently showed that non O blood group individuals had a significantly increased risk of venous thrombosis compared to O blood group individuals (i.e. non-BGAT protein secretion), and the risk of thrombosis is likely to be inversely correlated with the expression of ABH antigens [43], [44], [45]. Additionally, consistent with our findings among pregnant women, BGAT pQTLs were found to be associated with venous thromboembolism in non-pregnant population [17]. Such evidence suggests that BGAT protein could be a significant new drug target to prevent venous complication during pregnancy by regulating the expression of ABH antigen.

Hypertensive disorders of pregnancy constitute one of the leading causes of maternal and perinatal mortality worldwide [6], and have been reported closely relating to ABO blood groups [47]. Our study provided some evidence to further support the causal relationship between BGAT protein and gestational hypertension in the discovery analysis, although the findings did not pass Bonferroni correction tests in the validation test of Fenland and INTERVAL. It is worth mentioning that BGAT protein level and gestational hypertension across the three datasets showed similar effect estimates, suggesting that a higher level of BGAT might probably reduce the risk of gestational hypertension. Combining our finding for the unfavorable associations with venous complications, further studies need to confirm the protective effect on gestational hypertension, so as to inform antenatal care services to keep the BGAT protein level within a proper range.

Previous observational studies showed conflicted results for the association of ABO blood group and pre-eclampsia [48], [49], and our study provided little evidence for an association of BGAT protein level with pre-eclampsia. GDM is supposed to be closely related to T2D, and genome-wide association studies showed multiple lines of evidence pointed to the shared pathophysiology of GDM and T2D [50]. Recent studies also suggested that O blood group was independently associated with a higher risk of GDM [51], and AB blood group was considered as a protective factor [52]. Although we confirmed the causal relationship between BGAT protein level and T2D, there was little evidence supporting a causal relationship between BGAT protein level and GDM in our study. This could be due to insufficient power of the GDM GWAS, potential horizontal pleiotropy via other proteins, or suggests that the occurrence of T2D and GDM may be regulated by slightly different mechanisms.

### 4.3 Strength and limitation

Key strengths of this study include that 1) to the best of our knowledge, it is the first study to use MR to explore associations of BGAT protein with pregnancy outcomes; 2) Through LD clumping analysis, the selected pQTLs have low correlation with each other. This step is taken to ensure the independence of instruments and satisfy the Mendelian's second law; 3) comprehensive sensitivity analyses (e.g. positive control outcome, Steiger filtering, colocalization and proteome-wide MR) were applied to minimize potential bias due to violations of MR assumptions; 4) we investigated a range of adverse pregnancy outcomes in one paper.

Despite the listed strength, limitations do exist. First, this study may be vulnerable to insufficient statistical power, as the numbers of cases ranged between 478 to 30971 for our outcomes. Although we identified statistically robust association with venous complications (the outcome with the smallest N cases), true causal effects on other outcomes (e.g. GDM) may be too small to be detect under the current sample sizes. Second, FinnGen defined venous symptoms and haemorrhoids in pregnancy (O22 in ICD-10) by combining multiple subtypes (O22.0 to O22.9 in ICD-10). Given we used summary-level data, we could not explore associations of BGAT protein expression with those subtypes separately. Additionally, we noticed that both FinnGen and UK Biobank (field ID: 47270 and 47271) had very limited numbers of haemorrhoids cases based on their record linkage data of secondary care, even if this is expected to be a common disease in pregnancy [2]. This inconsistency could be because most patients did not have haemorrhoids severe enough to access to in-patient services. Thus, our results should be interpreted as the causal effect on severe venous conditions. Further studies based on self-report or primary care data are needed to investigated common diseases in pregnancy. Third, FinnGen included women with pre-existing disorders (e.g. diabetes and hypertension) into the case groups, and did not exclude never pregnant women from the controls [53]. But it seems unlikely that the results would be biased because of this. Fourth, our genetic instruments were obtained from non-pregnant population, and we have to assume that the SNP-exposure associations are not varied during pregnancy. Fifth, our MR results may still be prone to horizontal pleiotropy due to paternal or fetal genotype [54]. We did not adjust for them because of limited publicly available GWAS data. Finally, our results are limited to women of European descent, thus confirmation is required before generalizing the results from other populations. With the results limited to women of European descent, cohort differences are checked for protein assay technologies. Although the SOMA logic plan version differs from each cohort, which may lead to the different pQTL estimation in each dataset, our MR findings are replicated in three cohorts, therefore likely to be a true causal effect. We also acknowledge the needs for further studies, which will increase the generalizability of the findings to other ancestry groups, improve statistical power using large-scale GWAS of pregnancy outcomes, and triangulate evidence using observational data.

## 5 Conclusion

Our results suggest that plasma BGAT protein level has a causal effect on a higher risk of venous complications and hemorrhoids in pregnancy, which is unlikely to be influenced by horizontal pleiotropy. Our findings support prioritising BGAT protein as a potential drug target for T2D and the pregnancy complications.

## Code availability

The R scripts used in this study along with an informative user guide are now accessible on GitHub repository: github.com/geneinmylife/ABO-PREG.

## CRediT authorship contribution statement

**Yufang Bi:** Conceptualization, Funding acquisition, Writing – review & editing. **Jie Zheng:** Conceptualization, Methodology, Resources, Supervision, Writing – review & editing. **Yuqi Sun:** Formal analysis, Investigation, Visualization, Writing – original draft, Writing – review & editing. **Haonan Zheng:** Data curation, Formal analysis, Investigation, Software, Validation, Writing – original draft, Writing – review & editing. **Rongrong Gu:** Investigation, Writing – original draft. **Manqing Wang:** Investigation, Writing – original draft. **Xueyan Wu:** Writing – original draft. **Qian Yang:** Writing – original draft, Writing – review & editing. **Huiling Zhao:** Conceptualization, Supervision, Writing – review & editing.

## Declaration of Competing Interest

No potential conflict of interest was reported by the authors.

## Appendix A Supplementary material

Supplementary material 1. Strobe-MR checklist

.

Supplementary material 2. Supplementary tables (TableS1-S12)
