# Supplementary material for: The effect of histo-blood group ABO system transferase (BGAT) on pregnancy related outcomes：A Mendelian randomization study
Source: Comput Struct Biotechnol J. 2024 Apr 29;23:2067–75. doi: 10.1016/j.csbj.2024.04.040 (PMC11126538; doi:10.1016/j.csbj.2024.04.040)
Supplement: Supplementary file 1 — Supplementary material 1. Strobe-MR checklist [file mmc1.docx]

# Strobe-MR checklist

| **Item** | **Complete/location** |
| --- | --- |
| 1. **Title and Abstract:** "Mendelian randomization" is named both in the title and the abstract | Mendelian randomization been mentioned in the title. |
| **Introduction** |  |
| 1. **Background:** Explain the scientific background and rationale for the reported study. Is causality between exposure and outcome plausible? Justify why MR is a helpful method to address the study question. | Concept of Mendelian randomization and specific request for Mendelian randomization were explained in the third paragraph of the introduction. |
| 1. **Objectives:** State specific objectives clearly, including pre-specified causal hypotheses (if any). | The causal question has been stated in the fourth paragraph of the introduction. |
| **Methods** |  |
| 1. **Study design and data sources:** Present key elements of study design early in the paper. Consider including a table listing sources of data for all phases of the study. For each data source contributing to the analysis, describe the following:   a) Describe the study design and the underlying population from which it was drawn. Describe also the setting, locations, and relevant dates, including periods of recruitment, exposure, follow-up, and data collection, if available.  b) Give the eligibility criteria, and the sources and methods of selection of participants.  c) Explain how the analyzed sample size was arrived at.  d) Describe measurement, quality and selection of genetic variants.  e) For each exposure, outcome and other relevant variables, describe methods of assessment and, in the case of diseases, the diagnostic criteria used.  f) Provide details of ethics committee approval and participant informed consent, if relevant. | All necessary information about the GWAS studies been used in this study have been described in the Materials and Methods section and Table 2.  The genetic predictor selection process has been described in in the Materials and Methods section “Genetic instruments selection of BGAT protein levels”. |
| 1. **Assumptions:** Explicitly state assumptions for the main analysis (e.g. relevance, exclusion, independence, homogeneity) as well assumptions for any additional or sensitivity analysis. | The Mendelian randomization assumptions have been described in Materials and Methods section “Colocalization analysis” and “Proteome-wide association analysis of BGAT instruments and MR of potential pleiotropic proteins on pregnancy outcomes”. |
| 1. **Statistical methods main analysis**   Describe statistical methods and statistics used.  a) Describe how quantitative variables were handled in the analyses (i.e., scale, units, model).  b) Describe the process for identifying genetic variants and weights to be included in the  analyses (i.e, independence and model). Consider a flow diagram.  c) Describe the MR estimator, e.g. two-stage least squares, Wald ratio, and related statistics.  Detail the included covariates and, in case of two-sample MR, whether the same covariate set was used for adjustment in the two samples.  d) Explain how missing data were addressed.  e) If applicable, say how multiple testing was dealt with. | a) Described in the Materials and Methods section and Table 1.  b) Described in the section "Genetic instruments selection of BGAT protein levels" within Materials and Methods.  c) Described in the section "MR analysis" within Materials and Methods.  e) Described in the section "MR analysis" within Materials and Methods. |
| 1. **Assessment of assumptions: Describe any methods used to assess the assumptions or justify their validity.** | Described in “Colocalization analysis” and “Proteome-wide association analysis of BGAT instruments and MR of potential pleiotropic proteins on pregnancy outcomes” section in the Materials and Methods. |
| 1. **Sensitivity analyses:** Describe any sensitivity analyses or additional analyses performed. | The Mendelian randomization sensitivity analyses have been described in “MR analysis”, “Colocalization analysis” and “Proteome-wide association analysis of BGAT instruments and MR of potential pleiotropic proteins on pregnancy outcomes” section of the Materials and Methods. |
| 1. **Software and pre-registration**   a) Name statistical software and package(s), including version and settings used.  b) State whether the study protocol and details were pre-registered (as well as when and  where). | a) All statistical software and settings used are described in the Materials and Methods section.  b) The analysis plan was described in the Materials and Methods section and Figure 1. |
| **Results** |  |
| 1. **Descriptive data**   a) Report the numbers of individuals at each stage of included studies and reasons for exclusion. Consider use of a flow-diagram.  b) Report summary statistics for phenotypic exposure(s), outcome(s) and other relevant variables (e.g. means, standard deviations, proportions).  c) If the data sources include meta-analyses of previous studies, provide the number of studies, their reported ancestry, if available, and assessments of heterogeneity across these studies. Consider using a supplementary table for each data source.  d) For two-sample Mendelian randomization:  i. Provide information on the similarity of the genetic variant-exposure associations between the exposure and outcome samples.  ii. Provide information on extent of sample overlap between the exposure and outcome data sources. | a) Information is given in the Materials and Methods section and Figure 1.  b) We described the detailed information of the summary statistics for our instruments in Materials and Methods section, Supplementary Tables S1-4 and Figure 2. Other summary data was provided in Supplementary Table 6-7.  d) We provide this information in Materials and Methods section. |
| 1. **Main results**   a) Report the associations between genetic variant and exposure, and between genetic variant and outcome, preferably on an interpretable scale (e.g. comparing 25th and 75th percentile of allele count or genetic risk score, if individual-level data available).  b) Report causal effect estimate between exposure and outcome, and the measures of uncertainty from the MR analysis. Use an intuitive scale, such as odds ratio, or relative  risk, per standard deviation difference.  c) If relevant, consider translating estimates of relative risk into absolute risk for a meaningful time-period.  d) Consider any plots to visualize results (e.g. forest plot, scatterplot of associations between genetic variants and outcome versus between genetic variants and exposure). | a) Genetic exposure associations have been reported in Supplementary Table 1-4.  b) The causal effect estimates between exposures and outcomes were listed in Table 1.  Our results were presented in terms of odds ratio and confidence intervals throughout the results section for binary outcomes.  d) We visualized the results in Figure 3 using forest plot. |
| 1. **Assessment of assumptions**   a) Assess the validity of the assumptions.  b) Report any additional statistics (e.g., assessments of heterogeneity, such as I2, Q statistic). | a) We assessed the validity using sensitivity analyses, weighted median approach. Results were presented in the Results section. |
| 1. **Sensitivity and additional analyses**   a) Use sensitivity analyses to assess the robustness of the main results to violations of the assumptions.  b) Report results from other sensitivity analyses (e.g., replication study with different dataset, analyses of subgroups, validation of instrument(s), simulations, etc.).  c) Report any assessment of direction of causality (e.g., bidirectional MR).  d) When relevant, report and compare with estimates from non-MR analyses.  e) Consider any additional plots to visualize results (e.g., leave-one-out analyses). | a) we reported the use of genetic colocalization and proteome-wide MR as additional approach to test for Mendelian randomization.  b) Results of replication study with different dataset, validation of instruments using positive control and Proteome-wide association analysis are described in Results.  c) Steiger filtering results were listed in Supplementary Table 5.  e) Regional plots were used in Figure 4 to visualize the results. |
| **Discussion** |  |
| 1. **Key results** | Discussion paragraph 1 |
| 1. **Limitations**   Discuss limitations of the study, taking into account the validity of the MR assumptions, other sources of potential bias, and imprecision. Discuss both direction and magnitude of any potential bias, and any efforts to address them. | Discussed in the “Strength and limitation” section |
| 1. **Interpretations**   a) Give a cautious overall interpretation of results considering objectives and limitations.  Compare with results from other relevant studies.  b) Discuss underlying biological mechanisms that could be modelled by using the genetic  variants to assess the relationship between the exposure and the outcome.  c) Discuss whether the results have clinical or policy relevance, and whether interventions  could have the same size effect. | a) Interpretation: Discussion paragraphs 1, 2, 3, 4, 5, 6; Comparison with other studies: Discussion section “Comparison with previous studies”.  b) Discussion paragraph 2, 4, 5, 6  c) Discussion paragraph 1, 2, 3, 4, 5, 6. |
| 1. **Generalizability:** | We have discussed the potential caveats in terms of generalizability of our findings in the Discussion section “Strength and limitation”. |
| 1. **Funding:** | We have reported all sources of funding in the “Acknowledgements” section. |
| 1. **Data and data sharing:** | We have provided the link/approach to access genetic data used in this study in the “Materials and Methods section” section. The scripts been used in this study was available in “Code availability” section |
| 1. **Conflicts of Interest:** | All authors have declared conflicts of interest. |
